# Long-Term Outcomes and Safety of Surgical Treatment Following Intracranial Electroencephalography Monitoring in Pediatric Epilepsy: A Single-Center Study

**DOI:** 10.7759/cureus.108118

**Published:** 2026-05-01

**Authors:** Takafumi Shimogawa, Takato Morioka, Kimiaki Hashiguchi, Nobuya Murakami, Nobutaka Mukae, Hiroshi Shigeto, Yasunari Sakai, Ayumi Sakata, Akira Nakamizo, Koji Yoshimoto

**Affiliations:** 1 Department of Neurosurgery, Graduate School of Medical Sciences, Kyushu University, Fukuoka, JPN; 2 Department of Neurosurgery, Hachisuga Hospital, Munakata, JPN; 3 Department of Neurosurgery, Hashiguchi Neuro Clinic, Fukuoka, JPN; 4 Department of Neurosurgery, Fukuoka Children's Hospital, Fukuoka, JPN; 5 Department of Neurology, Graduate School of Medical Sciences, Kyushu University, Fukuoka, JPN; 6 Department of Health Sciences, Faculty of Medical Sciences, Kyushu University, Fukuoka, JPN; 7 Department of Pediatrics, Graduate School of Medical Sciences, Kyushu University, Fukuoka, JPN; 8 Department of Clinical Chemistry and Laboratory Medicine, Kyushu University Hospital, Fukuoka, JPN

**Keywords:** intracranial electroencephalography, long-term seizure outcomes, pediatric epilepsy, pediatric epilepsy surgery, safety

## Abstract

Objective: Intracranial electroencephalography (iEEG) monitoring presents additional challenges in pediatric patients compared to adults due to factors such as smaller body size, increased surgical invasiveness, difficulty maintaining rest, higher sensitivity to pain, and a greater risk of electrode self-removal. However, long-term outcomes and complications following resective surgery after iEEG evaluation in children remain underreported. This study aimed to evaluate the long-term outcomes and safety of surgical treatment following iEEG monitoring in pediatric patients.

Methods: We retrospectively analyzed 24 pediatric patients (aged ≤15 years) who underwent surgical treatment after iEEG implantation between 1994 and 2024, with a minimum follow-up period of 5 years. Clinical characteristics, neuroimaging findings, details of iEEG monitoring, surgical procedures, epilepsy etiology, and seizure outcomes were reviewed.

Results: The mean age at epilepsy onset was 4.5 years (range, 0.0-12.0), and the mean age at surgery was 10.4 years (range, 1.8-14.0). The average interval from seizure onset to surgery was 6.0 years (range, 0.9-14.8). The iEEG modalities included subdural grid electrodes alone (15 patients), a combination of subdural and depth electrodes (eight patients), and depth electrodes alone (one patient). The mean duration of iEEG monitoring was 9.1 days (range, 2-14). Surgical procedures included focal cortical resection and/or lesionectomy in 18 patients (75.0%), anterior temporal lobectomy in five (20.8%), and multiple subpial transections (MST) alone in one (4.2%). MST, in addition to resection, was performed in six patients (25.0%). Thirteen patients (54.2%) achieved Engel class I or II outcomes. No perioperative complications related to iEEG implantation occurred, and no patients experienced permanent neurological deterioration. Blood transfusion was not required in any case. MEG-MRI (magnetoencephalography-magnetic resonance imaging) concordance was significantly associated with favorable seizure outcomes.

Conclusion: This study demonstrated a good seizure outcome rate of 54.2% in pediatric patients who underwent surgical treatment following iEEG monitoring. No major perioperative complications related to iEEG implantation were observed, and no patients experienced permanent neurological deterioration in this cohort. These findings suggest that with careful electrode selection, precise placement and fixation, and vigilant perioperative management, iEEG can be safely performed even in very young children. Furthermore, the presence of MEG clusters concordant with MRI lesions tended to be associated with better seizure outcomes after resection, suggesting a possible role of multimodal imaging data in individualized treatment planning.

## Introduction

Epilepsy treatment is centered on antiseizure medication (ASM), the current cornerstone of management. However, approximately 30% of patients continue to experience seizures despite appropriate ASM therapy and are classified as having drug-resistant epilepsy [1]. For these patients, epilepsy surgery serves as a critical therapeutic option [2]. Surgical treatment for focal epilepsy is effective in both adult and pediatric patients with drug-resistant epilepsy. Evaluation for surgical candidacy incorporates multiple modalities, including clinical assessment, long-term video electroencephalography (EEG) monitoring, magnetic resonance imaging (MRI), fluorodeoxyglucose-positron emission tomography (FDG-PET), magnetoencephalography (MEG), single photon emission computed tomography (SPECT), and neuropsychological and developmental assessments. When noninvasive investigations do not sufficiently localize the epileptogenic zone, particularly in MRI-negative cases or when functional cortical mapping is necessary, chronic intracranial electroencephalography (iEEG) is employed [3-6]. iEEG monitoring is an essential tool in neocortical epilepsy surgery and is well established for the localization of epileptogenic zones and functional mapping in both adults and children [7-9].

However, iEEG in pediatric patients presents additional challenges compared to adult patients, including smaller body weight, increased surgical invasiveness, difficulty maintaining rest, higher sensitivity to pain, and a greater risk of electrode self-removal [10]. The minimum age or body weight for a safe craniotomy remains unclear and depends on institutional expertise. Consequently, iEEG placement and management in pediatric patients are typically more complex, and long-term outcomes and complications in pediatric epilepsy cases undergoing resective surgery after iEEG evaluation remain underreported [7,11-15]. Only a few studies have reported outcomes, including Yang et al. [11] (n=137), Onal et al. [13] (n=35), and Bauman et al. [12] (n=15).

This study reviewed pediatric epilepsy cases at our institution who underwent surgical treatment following intracranial electrode placement and were followed for ≥5 years. The primary objective of this study was to evaluate the long-term outcomes, efficacy, and safety of surgical treatment following iEEG. The secondary objective was to analyze factors associated with seizure outcomes.

## Materials and methods

Clinical profiles included treatment course of epilepsy, age at surgery, interval from seizure onset to surgery, neuroimaging and EEG findings, the identified epileptogenic zone, surgical details, pathological results, and seizure outcomes. These data were retrospectively obtained from medical charts and institutional databases. Ages are presented in years, except for patients aged <2 years, whose ages are reported in months.

Description of the study population

**Figure 1:**
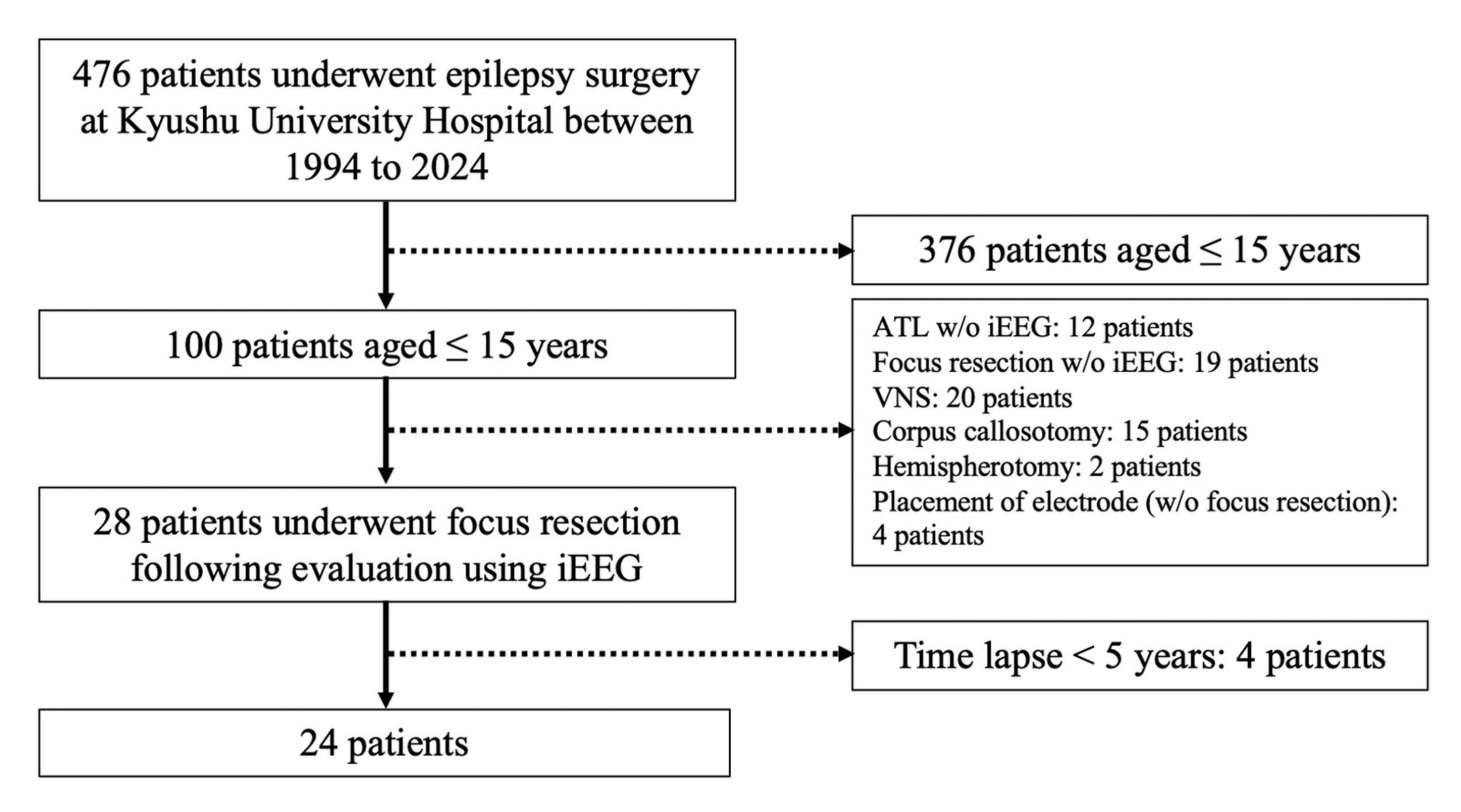
Flowchart of patient selection Abbreviations: iEEG, intracranial electroencephalography; ATL, anterior temporal lobectomy; w/o, without; VNS, vagus nerve stimulation

Between January 1994 and December 2024, 476 epilepsy surgeries were performed at Kyushu University Hospital. Among these, 100 patients were aged ≤15 years at the time of surgery. A total of 32 patients underwent iEEG implantation. Of these, four did not proceed to surgical treatment (three were diagnosed with multifocal epilepsy and one with generalized epilepsy), while 28 underwent surgical treatment following iEEG monitoring. A total of 24 patients with follow-up ≥5 years were included in this study (Figure 1).

Surgical technique for electrode implantation and iEEG monitoring

Before surgery, all patients underwent noninvasive evaluations, including long-term video EEG monitoring, MRI, FDG-PET, SPECT, MEG, neuropsychological assessments, and functional testing. MRI was performed using a 1.5-T system until 2006 and a 3.0-T system from 2007 onward. FDG-PET was performed throughout the study period. MEG was performed using a 37-channel MEG system until 2005 and a 306-channel whole-head MEG system from 2006 onward. MEG dipole estimation followed the protocols described previously [16]. SPECT data were excluded due to isotope variability. Neuropsychological testing (Wechsler Intelligence Scale for Children) was performed preoperatively in only 13 patients and postoperatively in four patients. However, owing to the long study period, a newer version of this test was used in some cases, resulting in a lack of consistency across assessments; therefore, these data were not included in the analysis.

Consistent with institutional policy, iEEG monitoring was indicated when MRI findings were negative or indicated widespread or multifocal abnormalities. Surgical indications and strategies, including implantation sites, electrode types, and numbers, were determined by a multidisciplinary epilepsy team composed of neurosurgeons, epileptologists, neurologists, pediatric neurologists, psychiatrists, and specialists in clinical laboratory and rehabilitation medicine. The indications for iEEG and the perioperative protocol remained consistent over the 30-year period (1994-2024), throughout which the senior author (TM) participated in all procedures.

Under general anesthesia, all patients underwent craniotomy and/or burr hole placement for implantation of grids, strips, and/or depth electrodes (Unique Medical Co., Ltd., Tokyo, Japan). After electrode implantation, the dura was closed in a watertight fashion, and the bone flap was replaced. Electrode wires were exteriorized through the skin incision and secured with a purse-string suture. Skull radiographs and computed tomography scans were obtained immediately after implantation. Postoperative monitoring was conducted in the epilepsy monitoring unit, where iEEG recording began immediately. Sedation was provided if patients had difficulty remaining at rest or were at increased risk of electrode self-removal.

The comprehensive epilepsy team reviewed iEEG data to delineate the epileptogenic zone. Surgical indication and procedure were determined by integrating noninvasive and invasive findings with functional and developmental assessments. Resection extent was guided by combined imaging and iEEG results. In cases where the epileptogenic zone involved or bordered eloquent cortex, multiple subpial transections (MST) were performed. All procedures were conducted by the same neurosurgical team (TM, KH, NM, NM, and TS) using a standardized technique and intraoperative electrocorticography.

Seizure outcome

Postoperative seizure outcomes were evaluated during outpatient or inpatient assessments, with the final follow-up outcome used for analysis. In principle, ASM dosage was maintained for one year after surgery without reduction. Seizure outcomes were classified using the Engel classification system.

Statistical analysis

Statistical analyses were conducted using Prism statistical software (GraphPad Software, Inc., La Jolla, California). Two-group comparisons were performed using Fisher’s exact test (for categorical variables) and the Mann-Whitney U test (unpaired non-parametric test); comparisons among three groups used the chi-square test. A p-value <0.05 was considered statistically significant.

## Results

Changes in strategies over time

The electrode implantation strategy evolved from the period between 1994 and 2015, when subdural grid electrodes alone were used (n=17), to the period between 2016 and 2024, when subdural grid and depth electrodes were used in combination (n=14). Stereoelectroencephalography (SEEG) was introduced in 2021 (n=1); however, the related case was excluded from the analysis owing to a follow-up of less than five years. In addition, until approximately 2000, iEEG was considered necessary even for mesial temporal lobe epilepsy. However, with improvements in the accuracy of noninvasive investigations and accumulating surgical experience, anterior temporal lobectomy (ATL) has since been performed without iEEG, except when lateralization of the epileptogenic focus is required.

Clinical characteristics and neuroimaging features of the study cohort

Table 1 summarizes the clinical characteristics and neuroimaging features. The cohort comprised 14 males and 10 females. The mean age at epilepsy onset was 4.5 years, and the mean age at surgery was 10.4 years, with a mean interval of 6.0 years between onset and surgery. MRI abnormalities were detected in 21 patients (87.5%). FDG-PET was conducted in 23 patients, revealing abnormalities in 19 (82.6%). MEG was performed in 11 of 24 patients; 13 did not undergo the examination owing to system unavailability or inability to maintain rest, as determined by the attending physician. MEG identified concordant clusters with MRI lesions in six patients (54.5%). Noninvasive evaluations localized the epileptogenic zone to the right hemisphere in 11 cases (45.8%) and to the left hemisphere in 13 cases (54.2%).

**Table 1:** Clinical information and neuroimaging features of the study cohort Abbreviations: MRI, magnetic resonance imaging; FDG-PET, fluorodeoxyglucose-positron emission tomography; MEG, magnetoencephalography

Variables	Values
Male sex, n (%)	14 (58.3)
Age at epilepsy onset, mean (range), years	4.5 (0.0–12.0)
Age at surgery, mean (range), years	10.4 (1.8–15.0)
Interval between epilepsy onset and surgery, mean (range), years	6.0 (0.9–14.8)
MRI abnormality, n (%)	
With abnormality	21 (87.5)
Without abnormality	3 (12.5)
FDG–PET abnormality, n (%) (23 cases were performed)	
With abnormality	19 (82.6)
Without abnormality	4 (17.4)
MEG abnormality, n (%) (11 cases were performed)	
Concordant localization with MRI lesion	6 (54.5)
Discordant localization with MRI lesion	5 (45.5)
Side of estimated epileptogenic zone, n (%)	
Right	11 (45.8)
Left	13 (54.2)

Characteristics of iEEG monitoring and surgical treatment

Table 2 details the iEEG monitoring and surgical procedures. Subdural grid electrodes alone were placed via craniotomy in 15 patients (62.5%), a combination of subdural grid and depth electrodes was employed in eight patients (33.3%), and depth electrodes alone were placed in one patient (4.2%), in whom a left-sided seizure onset zone was identified upon bilateral hippocampal depth electrode implantation, leading to ATL. The mean duration of iEEG monitoring was 9.1 days. Continuous antibiotic prophylaxis was administered throughout monitoring in all cases, while sedation was required in three patients (12.5%). Among all 32 patients who underwent iEEG implantation during the study period, including those who did not undergo surgical treatment, no transfusions were needed, and postoperative intracranial hemorrhages did not occur. There were no complications related to iEEG electrode placement, including cerebrospinal fluid leakage, infection, or permanent deficits. The presumed epileptogenic zone was localized to the frontal lobe in 11 patients (45.8%), the temporal lobe in seven (29.2%), the parietal lobe in three (12.5%), the occipital lobe in two (8.3%), and the parietotemporal region in one (4.2%). Six patients (25.0%) had a history of prior epilepsy surgeries, including focus resections, ATL, and vagus nerve stimulation (VNS). Following iEEG monitoring, surgical treatment consisted of focal cortical resection and/or lesionectomy in 18 patients (75.0%), ATL in five (20.8%), MST alone in one (4.2%), and MST combined with resection in six (25.0%). In the six patients who underwent MST combined with resection, the seizure onset zone involved the eloquent cortex, specifically the motor cortex in four cases and the language cortex in two. Therefore, MST was employed to preserve function rather than performing resection alone.

**Table 2:** Characteristics of intracranial EEG monitoring and surgical treatment Abbreviations: EEG, electroencephalography

Variables	Values
Types of implanted electrodes, n (%)	
Subdural electrodes implantation alone	15 (62.5)
Combined subdural electrode and depth electrode implantation	8 (33.3)
Depth electrodes implantation alone	1 (4.2)
Intracranial EEG monitoring periods, mean (range), days	9.1 (2–14)
Antibiotic prophylaxis administered, n (%)	24 (100)
Sedation administered, n (%)	3 (12.5)
Blood transfusion, n (%)	0 (0)
Complications, n (%)	0 (0)
Localization of estimated epileptogenic zone, n (%)	
Frontal	11 (45.8)
Temporal	7 (29.2)
Parietal	3 (12.5)
Occipital	2 (8.3)
Parieto–temporal	1 (4.2)
Prior other epilepsy surgeries, n (%)	6 (25.0)
Type of surgical treatment, n (%)	
Focal cortical resection and/or lesionectomy	18 (75.0)
Anterior temporal lobectomy	5 (20.8)
Multiple subpial transection	1 (4.2)
Multiple subpial transection in addition to surgical resection, n (%)	6 (25.0)

Etiology of epilepsy and seizure outcomes

Table 3 summarizes epilepsy etiology and seizure outcomes. Histopathological diagnoses were obtained in 23 cases, excluding those who underwent MST alone. Focal cortical dysplasia (FCD) type IIa was diagnosed in six cases (26.1%) and type IIb (4.3%) and type IIIc (4.3%) in one case each. Other findings included gliosis in seven patients (30.4%), hippocampal sclerosis (HS) in four (17.4%), tumors in two (8.7%), including pleomorphic xanthoastrocytoma and ganglioglioma, and tuberous sclerosis complex (4.3%) and meningioangiomatosis (4.3%) in one patient each. Eleven patients (45.8%) achieved Engel class I outcomes, two (8.3%) achieved class II, six (25.0%) were classified as class III, and five (20.8%) as class IV.

**Table 3:** Etiology of epilepsy and seizure outcomes Abbreviations: FCD, focal cortical dysplasia

Variables	Values
Etiology of epilepsy, n (%) (23 cases were diagnosed)	
FCD type IIa	6 (26.1)
FCD type IIb	1 (4.3)
FCD type IIIc	1 (4.3)
Gliosis	7 (30.4)
Hippocampal sclerosis	4 (17.4)
Tumor	2 (8.7)
Tuberous sclerosis complex	1 (4.3)
Meningioangiomatosis	1 (4.3)
Engel Classes, n (%)	
I	11 (45.8)
II	2 (8.3)
III	6 (25.0)
IV	5 (20.8)

Clinical characteristics associated with a good seizure outcome after surgical treatment

Table 4 compares patients with good outcomes (13 patients; Engel class I/II) and poor outcomes (11 patients; class III/IV). Sex distribution did not differ between groups. The mean age at epilepsy onset was 4.78 years in the good outcome group and 4.07 years in the poor outcome group. The mean age at surgery was 10.9 years and 9.8 years, with a mean interval from onset to surgery of 6.14 and 5.82 years, respectively.

**Table 4:** Clinical characteristics associated with seizure outcome * p < 0.05 Abbreviations: MRI, magnetic resonance imaging; FDG-PET, fluorodeoxyglucose-positron emission tomography; MEG, magnetoencephalography

Variables	Engel Class I/II	Engel Class III/IV	p-value
Sex, n			>0.999
Male	8	6	
Female	5	5	
Age at epilepsy onset, mean (range), years	4.78 (0.8–9.0)	4.07 (0.0–12.0)	0.628
Age at surgery, mean (range), years	10.9 (4.0–15.0)	9.80 (1.8–15.0)	0.542
Interval between epilepsy onset and surgery, mean (range), years	6.14 (1.0–11.0)	5.82 (0.9–14.8)	0.830
MRI abnormality, n (%)			0.576
With abnormality	12 (92.3)	9 (81.8)	
Without abnormality	1 (7.7)	2 (18.2)	
FDG–PET abnormality, n (%) (performed in 23 cases)			>0.999
With abnormality	10 (83.3)	9 (81.8)	
Without abnormality	2 (16.7)	2 (18.2)	
MEG abnormality, n (%) (performed in 11 cases)			0.015*
Concordant localization with MRI lesion	5 (100)	1 (16.7)	
Discordant localization with MRI lesion	0 (0)	5 (83.3)	
Side of estimated epileptogenic zone, n (%)			0.682
Right	5 (38.5)	6 (54.5)	
Left	8 (61.5)	5 (45.5)	
Localization of estimated epileptogenic zone, n (%)			-
Frontal	5 (38.5)	6 (54.5)	
Temporal	4 (30.8)	3 (27.3)	
Parietal	3 (23.1)	0 (0)	
Occipital	1 (7.7)	1 (9.1)	
Parieto–temporal	0 (0)	1 (9.1)	

MRI abnormalities were identified in 12 patients (92.3%) in the good outcome group and in nine patients (81.8%) in the poor outcome group. FDG-PET abnormalities were present in ten (83.3%) and nine patients (81.8%), respectively. Concordant MEG clusters corresponding to MRI lesions were observed in all patients (100%) in the good outcome group but in only one patient (16.7%) in the poor outcome group (p = 0.015). Right-sided epileptogenic zones were identified in five patients (38.5%) in the good outcome group and in six patients (54.5%) in the poor outcome group. In the good outcome group, presumed epileptogenic lesions were located in the frontal lobe in five patients (38.5%), in the temporal lobe in four (30.8%), in the parietal lobe in three (23.1%), and in the occipital lobe in one (7.7%). In the poor outcome group, the frontal lobe was involved in six patients (54.5%), the temporal lobe in three (27.3%), and the occipital lobe (9.1%) and parietotemporal lobe (9.1%) in one patient each.

Table 5 summarizes surgical methods and etiological factors. Prior epilepsy surgery was performed in two patients (15.4%) in the good outcome group (one focus resection and one VNS) and in four patients (36.4%) in the poor outcome group (three focus resections and one ATL). Regarding electrode placement, in the good outcome group, seven patients (53.8%) received subdural grid electrodes alone, five (38.5%) received both subdural grid and depth electrodes, and one (7.7%) received depth electrodes alone. In the poor outcome group, eight patients (72.7%) received subdural grid electrodes alone, and three (27.3%) received both electrode types. The mean iEEG monitoring duration was 10 days in the good outcome group and eight days in the poor outcome group. Sedation was administered during iEEG monitoring in one patient (7.7%) and two patients (18.2%), respectively. Regarding the surgical procedures, focal cortical resection and/or lesionectomy was performed in seven patients (53.8%), ATL in five (38.5%), and MST alone in one (7.7%) in the good outcome group. In the poor outcome group, focal cortical resection and/or lesionectomy was performed in eight patients (72.7%) and ATL in three (27.3%). MST combined with resection was performed in one patient (7.7%) in the good outcome group and in five patients (45.5%) in the poor outcome group. Histopathological diagnoses in the good outcome group included FCD type IIb in one patient (8.3%), FCD type IIIc in one patient (8.3%), gliosis in three patients (25.0%), HS in four patients (33.3%), tumors in two patients (16.7%), and meningioangiomatosis in one patient (8.3%). In the poor outcome group, FCD type IIa was observed in six patients (54.5%), gliosis in four (36.4%), and tuberous sclerosis complex in one (9.1%).

**Table 5:** Characteristics of surgical methods and etiology of epilepsy associated with good seizure outcome Abbreviations: EEG, electroencephalography; FCD, focal cortical dysplasia

Variables	Engel Class I/II	Engel Class III/IV	p-value
Prior other epilepsy surgeries, n (%)	2 (15.4)	4 (36.4)	0.357
Types of implanted electrodes, n (%)			0.494
Subdural electrodes implantation alone	7 (53.8)	8 (72.7)	
Combined subdural electrode and depth electrode implantation	5 (38.5)	3 (27.3)	
Depth electrodes implantation alone	1 (7.7)	0 (0)	
Intracranial EEG monitoring periods, mean (range), days	10.0 (7–14)	8.0 (2–14)	0.163
Antibiotic prophylaxis administered, n (%)	13 (100)	11 (100)	-
Sedation administered, n (%)	1 (7.7)	2 (18.9)	0.576
Blood transfusion, n (%)	0 (0)	0 (0)	-
Complications, n (%)	0 (0)	0 (0)	-
Type of surgical treatment, n (%)			0.494
Focal cortical resection and/or lesionectomy	7 (53.8)	8 (72.7)	
Anterior temporal lobectomy	5 (38.5)	3 (27.3)	
Multiple subpial transection	1 (7.7)	0 (0)	
Additional multiple subpial transection, n	1 (7.7)	5 (45.5)	0.061
Etiology of epilepsy, n (%) (23 cases were diagnosed)			
FCD type IIa	0 (0)	6 (54.5)	-
FCD type IIb	1 (8.3)	0 (0)	
FCD type IIIc	1 (8.3)	0 (0)	
Gliosis	3 (25.0)	4 (36.4)	
Hippocampal sclerosis	4 (33.3)	0 (0)	
Tumor	2 (16.7)	0 (0)	
Tuberous sclerosis complex	0 (0)	1 (9.1)	

## Discussion

iEEG implantation and management are more challenging in pediatric patients than in adult patients. Although several studies have reported long-term outcomes and complications after surgery following iEEG in pediatric patients, available data remain limited [7,11-15]. Harvey et al. [17] reported that 22.7% of 543 pediatric patients (<18 years of age) with drug-resistant epilepsy underwent surgery following iEEG across 20 centers. Wang et al. [18] analyzed the Healthcare Cost and Utilization Project Kids' Inpatient Database (2000-2012) and found that only 2.56% of pediatric patients (≤18 years of age) underwent iEEG. In Japan, Saito et al. [19] reported that, based on a health claims database, among 9,279 patients (<18 years of age) with epilepsy in 2018, only 28 underwent epilepsy surgery, and just four underwent resective surgery following iEEG. To date, no studies conducted in Japan have specifically examined long-term outcomes or complications after surgical treatment following iEEG in pediatric patients.

In our cohort of pediatric patients (≤15 years of age) who underwent surgical treatment after iEEG and were followed for ≥5 years, 54.2% achieved favorable outcomes (Engel class I/II). Although four cases were excluded from the long-term outcome analysis, their short-term outcomes were as follows: two patients achieved Engel class I, and two were classified as class IV. Yang et al. [11] reported a 72.2% rate of Engel class I/II outcomes in 137 pediatric patients (mean implantation age: 12.6 ± 3.8 years) who underwent iEEG with subdural grid and depth electrodes. Onal et al. [13] reported a 69% Engel class I/II outcome rate in 35 pediatric patients (≤19 years of age) using either subdural grid electrodes alone or in combination with depth electrodes. Bauman et al. [12] reported a 60% Engel class I/II outcome rate in 15 pediatric patients (<19 years of age) with extratemporal lobe epilepsy (ETLE) undergoing invasive monitoring with subdural grid electrodes alone. Although our long-term outcomes were favorable, the slightly lower success rate may reflect specific characteristics of our cohort. One contributing factor is the high proportion of cases with epileptogenic zones involving eloquent cortex, requiring more frequent use of MST alongside resection.

In a study by Harvey et al. [17] of 543 pediatric epilepsy surgery patients, MST was used in only 0.6% of cases. In contrast, MST was performed in six cases (25%) in our series. Although the difference in MST frequency between outcome groups was not statistically significant, its higher use may have influenced overall results. Another possible explanation is the predominance of ETLE in our cohort. Harvey et al. [17] reported temporal lobe epilepsy (TLE) in 48.3% of patients, whereas in our study, TLE accounted for only 29.2%. Consistent with prior literature in both adults and older children, surgical outcomes for ETLE are generally less favorable than for TLE [20,21]. We also evaluated outcomes in 31 patients who underwent surgical treatment without iEEG (19 focal resections and 12 ATLs). Among these patients, 14 (45.2%) achieved Engel class I outcomes, four (12.9%) class II, 10 (32.3%) class III, and three (9.7%) class IV. There was no significant difference in favorable outcomes (Engel class I/II) compared with those who underwent surgical treatment following iEEG monitoring (p=0.707). This suggests that iEEG may help achieve outcomes comparable to those of standard surgical cases, even in more complex or difficult cases. In addition, iEEG provided important clinical information that directly influenced management decisions. In this study, iEEG was performed in a total of 32 patients. In four patients, iEEG enabled diagnoses that could not be established through noninvasive evaluations, thereby avoiding unnecessary surgical treatment. In addition, in one patient with TLE in whom lateralization could not be determined noninvasively, iEEG successfully identified the laterality. Furthermore, iEEG localized the seizure onset zone within the eloquent cortex in six patients, allowing for tailored surgical strategies. In these cases, MST was performed instead of resection, enabling preservation of neurological function.

In our study, among six patients with prior epilepsy surgery, two achieved favorable outcomes, and four had unfavorable outcomes. Two patients in the poor outcome group subsequently underwent additional interventions: one hemispherotomy and one resection guided by intraoperative electrocorticography; both subsequently achieved Engel class I outcomes. For patients with persistent seizures following initial surgery, further evaluation with iEEG, additional focal resection, or palliative approaches should be considered [22,23].

Following iEEG implantation, complication rates have been reported to be higher in younger patients [12]. Major perioperative complications in pediatric patients include infections (up to 8.6%), cerebrospinal fluid (CSF) leakage (up to 31.3%), subdural or intraparenchymal hematomas (up to 25.7%), and cerebral edema (up to 14.2%) [7,12-15], with occasional need for blood transfusion [12,13,15]. Minor complications include hypertrophic scarring, alopecia, bone defects, hydrocephalus, and electrolyte disturbances such as hyponatremia. Four cases were excluded from the long-term outcome analysis; however, no major perioperative complications related to iEEG implantation were observed, and none of the 28 patients who underwent surgery experienced permanent neurological deterioration. No patients required transfusion during or after surgery. We also evaluated complications in 31 patients who underwent surgical treatment without iEEG. In the non-iEEG group, one patient developed transient motor weakness suggestive of supplementary motor area syndrome, and another developed transient aphasia. These findings suggest that iEEG use may help reduce surgical complications without compromising overall procedural safety. To enhance safety, several surgical strategies were implemented at our institution. Grid electrodes with ≤15 contacts were used to reduce intracranial pressure whenever feasible. To prevent displacement or overlap, which can result in compression and hemorrhage, each grid was sutured together using 4-0 nylon. The dura was closed watertight to minimize CSF leakage. During monitoring, daily assessments were conducted by physicians, clinical technologists, and nurses to detect CSF leakage. Vital signs, skin condition, and fever were reviewed and communicated daily within the care team. Continuous antibiotic prophylaxis (cefazolin) was administered. Steroids were not used for the prevention of cerebral edema. Until 2022, total head shaving was standard practice; thereafter, partial shaving has been adopted. Hair washing was performed once during the first monitoring week when possible. Notably, iEEG monitoring was safely completed without sedation in the youngest patient (one year and nine months), demonstrating feasibility in very young children.

We examined factors associated with seizure outcomes by comparing the good and poor outcome groups. Although shorter epilepsy duration is typically correlated with improved outcomes [24], no significant difference was observed between groups. Interestingly, the presence of MEG clusters concordant with MRI lesions tended to be associated with better seizure outcomes after resection, supporting the utility of MEG for localizing epileptogenic zones [16,25]. MRI and FDG-PET abnormalities were more frequently observed in the good than the poor outcome group, consistent with previous reports [24,26,27]. As surgical outcomes for pediatric ETLE are often less favorable than those for TLE [20], the higher proportion of TLE cases with HS in the good outcome group is noteworthy. No significant differences in seizure control were observed between patients receiving subdural grid electrodes alone and those undergoing combined subdural grid and depth electrode implantation. We also analyzed outcomes by era: 1994-2015, when only subdural grid electrodes were used, and 2016-2024, when subdural grid and depth electrodes were combined. From 1994 to 2015, outcomes were Engel class I in six patients, class II in two, class III in five, and class IV in three, yielding a 50% favorable outcome rate (Engel class I/II). From 2016 to 2024, outcomes were Engel class I in five patients, class III in one, and class IV in two, corresponding to a 62.5% favorable outcome rate. Overall, outcomes were better during the period in which subdural grid and depth electrodes were used in combination. Depth electrodes play a critical role in identifying epileptogenic zones in deep or mesial structures, including the insula, mesial temporal lobe, and interhemispheric regions [28]. Given the higher prevalence of extratemporal and FCD-related epilepsy in pediatric populations [17,29], individualized implantation strategies incorporating depth electrodes are essential [29]. Although SEEG was not utilized in this series, it represents a promising minimally invasive alternative, particularly for widespread or deep-seated epileptogenic foci, as well as those requiring reoperation [30].

This study has several limitations. First, it was conducted at a single center and involved a relatively small cohort of patients, underscoring the need for larger, multicenter studies to confirm and expand upon these findings. In addition, postoperative seizure outcomes were assessed during outpatient or inpatient follow-up, with the final available outcome used for analysis. However, as the timing of the final follow-up varied among patients, the potential for selection bias cannot be excluded. Second, the study period extended from January 1994 to December 2024. Over the 30-year period, the indications for iEEG remained consistent; however, the electrode implantation strategy and imaging modalities evolved. These changes may have influenced surgical outcomes. In addition, with advances in diagnostic techniques, iEEG has been increasingly adopted. This is likely attributable to the introduction of depth electrodes and SEEG, which have enabled evaluation of complex and previously intractable cases that were difficult to assess using subdural grid electrodes alone. Third, the presence of MEG clusters concordant with MRI lesions tended to be associated with better seizure outcomes after resection; however, because MEG was performed in only 11 of 24 patients, selection bias related to indication cannot be excluded. Fourth, the long-term developmental outcomes following surgery remain unclear. In pediatric patients, the goals of treatment extend beyond seizure reduction to include prevention of cognitive and developmental stagnation or regression resulting from persistent epileptic activity or the adverse effects of ASMs [2]. Further studies will be necessary to elucidate the relationship between the underlying etiology of epilepsy and postoperative improvements in cognitive function.

## Conclusions

This study demonstrated that the rate of good outcomes in pediatric patients undergoing surgical treatment following iEEG monitoring was 54.2%. No major perioperative complications related to iEEG implantation were observed, and no patients experienced permanent neurological deterioration in this cohort. Careful electrode selection, meticulous placement and fixation, and close perioperative management may help ensure procedural safety even in very young children. Additionally, the presence of MEG clusters concordant with MRI lesions tended to be associated with better seizure outcomes after resection, suggesting a possible role of multimodal imaging data in individualized treatment planning.
